# P(VDF-TrFE)/PMMA Blended Films with Enhanced Electrowetting Responses and Superior Energy Storage Performance

**DOI:** 10.3390/polym11030526

**Published:** 2019-03-20

**Authors:** Jiayun Chen, Xiaoying Xiong, Qilong Zhang, Lingling Shui, Shitao Shen, Hui Yang, Zhicai Zhu, Fang Zhang

**Affiliations:** 1 School of Materials Science and Engineering, State Key Lab Silicon Mat, Zhejiang University, Hangzhou 310027, China; 21626057@zju.edu.cn (J.C.); 21626025@zju.edu.cn (X.X.); yanghui@zju.edu.cn (H.Y.); 21726096@zju.edu.cn (Z.Z.); 2 National Center for International Research on Green Optoelectronics & South China Academy of Advanced Optoelectronics, South China Normal University, Guangzhou 510006, China; shuill@m.scnu.edu.cn (L.S.); shitao.shen@guohua-oet.com (S.S.); 3 Zhejiang California International NanoSystems Institute, Zhejiang University, Hangzhou 310058, China; Zhang_fang2121@sina.com

**Keywords:** electrowetting, energy storage, dielectric constant, hydrophobicity

## Abstract

P(VDF-TrFE) (vinylidene fluoride-co-trifluoroethylene)/PMMA (PVT/PMMA) blended films synthesized through a facile solution-blending method show outstanding performance for practical electrowetting and energy storage applications. The van der Waals forces and dipolar interactions in neighboring P(VDF-TrFE) and PMMA chains, together with the suppressed free volume (or defect) are critical to the significantly-enhanced electrical properties. Typical, Teflon-covered P(VDF-TrFE)/PMMA blended film exhibits a high dielectric constant of 13 with low dielectric loss (~0.05) at 100 Hz and a large initial contact angle of 122°. Its electrowetting response with a contact angle modulation of 50° in air and low contact angle hysteresis demonstrate that it is promising for low-voltage electrowetting applications. Furthermore, with an energy density of 11.8 J/cm^3^, approximately double that of pure P(VDF-TrFE), PVT/PMMA blended films containing 20 wt % PMMA turn out to be superior materials for energy storage applications, due to their significantly-enhanced polarization and reduced remnant polarization.

## 1. Introduction

**Figure 1:**
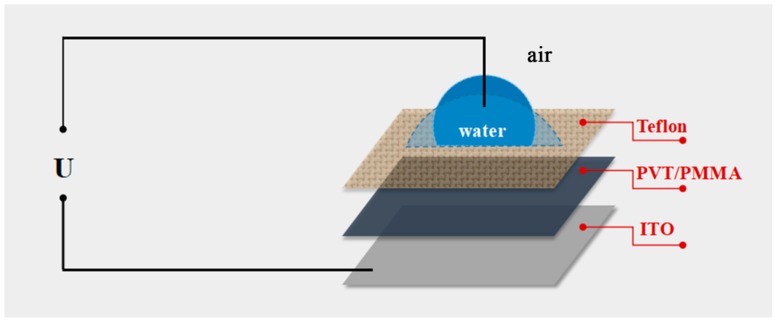
Schematic of the electrowetting experiment setup in the air ambient condition: ITO as the electrode; PVT/PMMA as the dielectric layer; Teflon as hydrophobic layer; water as the aqueous droplet.

EWOD [1] (electrowetting-on-dielectric) is a versatile technique based on an apparent contact angle modulation controlled by the applied electric field, which has gained attention in micro-scale systems [2,3], including liquid lenses [4,5], optical waveguides [6], lab-on-a-chip devices [7,8], electronic displays [9,10,11], digital microfluidics [12], etc. General EWOD devices consist of an electrode, a conductive aqueous droplet, a dielectric layer with hydrophobic and insulating properties deposited on the electrode, and an external voltage (shown in Figure 1). The relationship between the change of surface wettability associated with interfacial surface energy [13] and the applied voltage can be explained by the Young−Lippmann Equation (1):

(1) $$\cos\left(\theta \right)=\cos\left(\theta_{0}\right)+\frac{\varepsilon_{0}\varepsilon }{2{d\gamma }_{\mathrm{LV}}}U^{2}$$

where *θ* is the contact angle of the liquid droplet under applied voltage *U*, $\theta_{0}$ is the initial contact angle without voltage applied, $\varepsilon_{0}$ is the permittivity of free space, ε is the dielectric constant of the dielectric layer, d is the dielectric thickness, and $\gamma_{LV}$ is the liquid-vapor interfacial energy. This equation clearly shows that the ideal electrowetting phenomenon should be realized under minimum voltage with the maximum contact angle modulation. In order to cut down the power consumption, it requires a high dielectric constant (ε), low interfacial energy ($\gamma_{LV})$, generally realized in an oil ambient condition, small dielectric thickness (d), and large initial contact angle ($\theta_{0})$. In addition, contact angle hysteresis (CAH) is widely known to adversely prevent the droplet from rapid and accurate response to the applied voltage during the electrowetting process. As a result, more effort is required to combine the high dielectric constant and contact angle with large tunable modulation, as well as minimal hysteresis. Therefore, most researchers combined two separate materials for dielectric and hydrophobic layers. Typically, the dielectric materials for EWOD studies are metal oxides or nitrides with high permittivity, including Al_2_O_3_ [14,15], Ta_2_O_5_ [16,17,18], ZrO_2_, TiO_2_ [19], and Si_3_N_4_/SiO_2_ [20]. However, their synthesis requires high temperatures along with complicated techniques like atomic layer deposition (ALD), chemical vapor deposition (CVD), sputtering, or electrochemical anodization. Furthermore, many researchers have used polymer dielectrics, such as polyimide [21], parylene-C, and parylene-N [22], to simplify the fabrication and reduce the processing cost. However, these polymers are restricted by the relatively low dielectric constant (approximately 2~4), which leads to a high switching voltage, usually at least 200 V, to obtain a significant contact angle modulation [23].

On the contrary, poly(vinylidene fluoride) polymer and PVDF-based copolymers, widely known to exhibit high energy storage with high polarization [24,25,26,27,28,29], show promising properties as the most suitable dielectric for electrowetting applications. The recent study of the electrowetting response on Teflon-covered PVDF-HFP insulator (ε_eff_ ≈ 6) showed its application potential in EWOD devices, but still, there is a problem with the hysteresis ΔU of 17 V for DC voltage [30], mainly due to the remnant polarization induced by the nature of ferroelectric polymers. In this paper, ferroelectric polymer P(VDF-TrFE) (vinylidene fluoride-co-trifluoroethylene) was chosen as the dielectric material for its extremely high permittivity. Linear PMMA polymer was introduced into P(VDF-TrFE) matrix in order to minimize the ferroelectric effect of P(VDF-TrFE) and thus increase the reversibility. In addition, dynamic hysteresis is also associated with the friction force between water droplets and the insulator surface [31,32]. To minimize this interaction, Teflon was selected to cover the dielectric layer. Combined with a layer of Teflon, the blended film presents an obvious contact angle modulation of 50° in air with large initial contact angle and low contact angle hysteresis (ΔU ≈ 10 V). On the basis of theoretical calculations, such blended film is a promising dielectric insulator for electrowetting applications with low energy consumption and fast response speed.

Furthermore, poly(vinylidene fluoride) P(VDF)-based polymer and copolymer exhibiting high polarization also demonstrate promising properties for high energy storage systems. As we know, the energy storage of dielectric materials is mainly determined by the dielectric constant and breakdown strength. The introduction of inorganic fillers with a high dielectric constant is regarded as the most effective method to improve the energy storage density of PVDF-based polymer and copolymers further [26,27,28,33,34]. However, a high-volume fraction of ceramic fillers is normally required to achieve the high energy storage density, which will inevitably worsen the flexibility and processability of the composite film, restricting the application in flexible electronics. Thus, many researchers have been focusing on the chemical modification of polymer or polymer-based composites for flexible energy storage application. Polymer blending is an effective method because it combines both the advantages of base polymer and the additional polymer. For instance, a higher energy density of about 11.5 J/cm^3^ is obtained in P(VDF/CTFE) blends in contrast to about 9 J/cm^3^ in P(VDF-TrFE-CFE) terpolymers [29]. Therefore, we also investigated the energy density of PVT/PMMA blended films. It is interesting to note that the breakdown strength of PVT/PMMA blended films is significantly higher than both pure PVT and PMMA, due to the van der Waals forces and dipolar interactions strengthening the interface interactions. The energy density of PVT/PMMA containing 20 wt % PMMA reached up to 11.8 J/cm^3^, approximately double that of pure P(VDF-TrFE), originating from the significantly-enhanced polarization and reduced remnant polarization. Herein, a significantly promising blended film with outstanding performance for practical electrowetting and high energy-density applications has been successfully synthesized through a facile solution-based method.

## 2. Materials and Methods 

### 2.1. Materials

P(VDF-TrFE) (50:50 mol%, Mw: 400–600 10^3^ g/Mol) powder was provided by Wuhan CYMENES Technology Co. Ltd (Wuhan, China). The poly (methyl methacrylate) (PMMA) with average molecular weight of about 120,000 by GPC was supplied by Sigma-Aldrich (Shanghai, China). Teflon AF 1600 was purchased from DuPont, Wilmington, Delaware, USA. Fluorinert FC-43 (Sigma-Aldrich) was used as the solvent. N,N-dimethylformamide (DMF) was purchased from Aladdin Industrial Corporation (Shanghai, China).

### 2.2. Fabrication of PVT/PMMA Blended Films

First, P(VDF-TrFE) (vinylidene fluoride-co-trifluoroethylene) powder was dissolved in DMF under vigorous stirring. At the same time, various weights of PMMA were dissolved in DMF under vigorous stirring, controlling the solute weight/solvent volume to 1 g/10 mL. Six different compositions of the blended films were employed with the following PMMA contents: 20 wt % (PVT/PMMA1), 27 wt % (PVT/PMMA2), 33 wt % (PVT/PMMA3), 50 wt % (PVT/PMMA4), 60 wt % (PVT/PMMA5), 67 wt % (PVT/PMMA6). After 6 h, these two polymer solutions were mixed, and an additional small amount of DMF was added. Finally, a solution with the polymers/solvent ratio of 1 g/15 mL was prepared. Then, the solution was vigorously stirred for another 12 h before spin coating. Tin oxide (ITO)-coated glass substrates (Sigma-Aldrich, Shanghai, China) were cleaned in an ultrasonic bath containing acetone followed by ethyl alcohol for 10 min each. The solution containing different content of PMMA was applied on ITO by spin coating, at 780 rpm for 18 s first and then 1700 rpm for 40 s. The coating process was repeated three times to achieve a desired film thickness (1.0–5.0 μm), after which a layer of Teflon AF solution (1 wt % in Fluorinert FC-43) was coated on the top. The rest of the solution was cast onto glass slides for further characterization. The films were dried at 60 °C for 24 h to remove the residual solvent.

### 2.3. Characterization

The morphology of the surface and the cross-sections of the films freeze-fractured in liquid nitrogen before testing were observed using a field emission scanning electron microscopy (FESEM, S-4800, Hitachi Ltd, Tokyo, Japan) and atomic force microscopy (AFM, MultiMode, VEECO Co., Billerica, Massachusetts, USA). The phase transition and crystallinity of the films were evaluated by XRD (EMPYREAN, PANalytical Co., Almelo, The Netherlands) with Cu Kα radiation and a 2θ scanning range of 10–80°. The chemical structure was characterized using Fourier transform infrared spectrometer (FT-IR; VERTEX-70, Bruker, Ettlingen, Germany) in a spectral range of 4000–400 cm^−1^. The DC breakdown strength test was carried out on a breakdown voltage instrument (CS2674AX, Nanjing Changsheng Instrument Co. Ltd., Nanjing, China) in silicone oil at room temperature. The dielectric properties and D-E loops of the films were measured with 50-nm Pt electrodes deposited on both sides using a mask with 4 mm-diameter eyelets. The static contact angle (CA) was observed by OCA20 (Dataphysics, Stuttgart, Germany). The thickness of the insulating layers was measured using a profilometer (DEKTAK-XT). Typically, the thickness of PVT/PMMA1/Teflon was 2.3 ± 0.01 μm with PVT/PMMA1 of 2.2 ± 0.01 μm and Teflon AF of 0.1 ± 0.01 μm. The AC voltage signal was generated with a function generator (AFG1062) and further amplified using an OPAMP (ATA-2042) manufactured by Agitek. PSW 800–1.44 (Guwei, Taipei, Taiwan) was employed as a voltage source for DC electrowetting study. EW response was tested using a 2-μL water droplet dispensed on the dielectric surface in air.

## 3. Results and Discussion

### 3.1. Morphology and Structure

**Figure 2:**
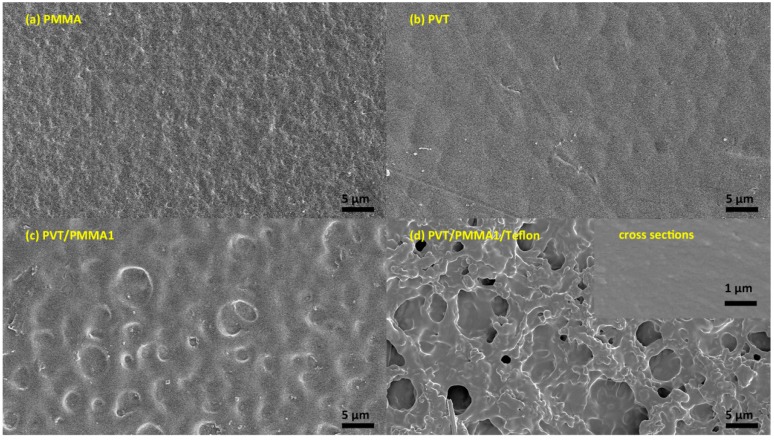
SEM images of (**a**) pristine PMMA film, (**b**) pristine P(VDF-TrFE) (vinylidene fluoride-co-trifluoroethylene) film, (**c**) PVT/PMMA1 blended film, and (**d**) PVT/PMMA1 blended film covered with a layer of Teflon. The inset of (**d**) is the freeze-fractured cross-section of the PVT/PMMA1/Teflon film.

**Figure 3:**
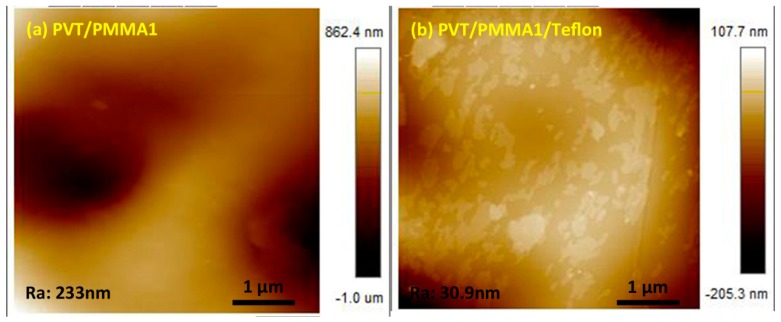
AFM image of (**a**) PVT/PMMA1 and (**b**) PVT/PMMA1/Teflon blended films.

In order to support our findings with a contact angle of over 120° and low contact angle hysteresis (CAH), scanning electron microscopy (SEM) and atomic force microscopy (AFM) were used to analyze the morphology. As shown in Figure 2a,b, the surface of pristine PMMA and P(VDF-TrFE) was smooth. In contrast, hierarchical arrays, tending to form spherulites with some orientation, are visible on the surface of PVT/PMMA1 composites (Figure 2c). Such a morphology might be caused by the effect on crystallization of P(VDF-TrFE) induced by PMMA. Figure 2d shows that the fibers of Teflon are randomly distributed; this kind of morphology is favorable to trap air and thus improve the contact angle. No pores or cracks could be found in the surface of pure P(VDF-TrFE) film, pure PMMA film, nor PVT/PMMA1 blended films, and the freeze-fractured cross-section of the PVT/PMMA1/Teflon blended film revealed the homogeneity and uniformity of polymer films. AFM provided detailed information about the surface structure. As shown in Figure 3, the film covered with a layer of Teflon was smoother than the PVT/PMMA1 film, with a roughness of 30.9 nm and 233 nm, respectively, which is critical to lower contact angle hysteresis [27]. 

**Figure 4:**
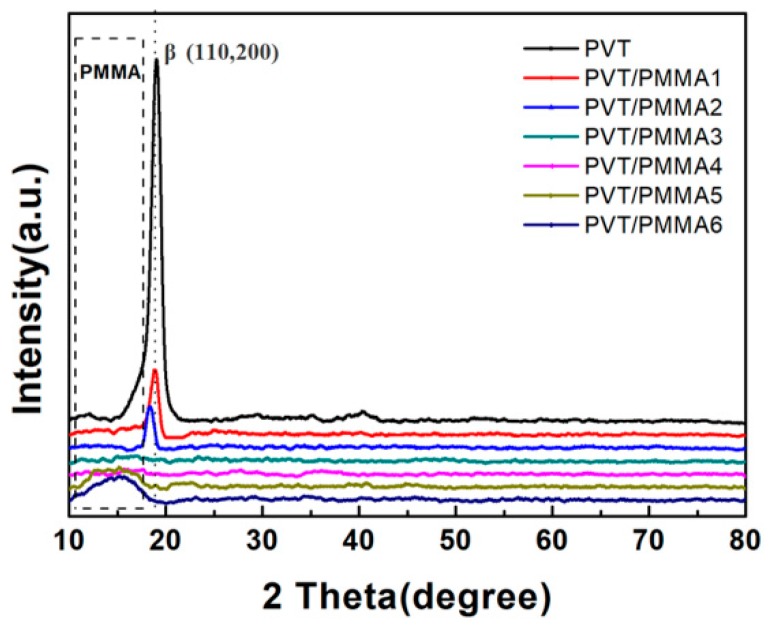
XRD patterns of PVT/PMMA composites with different contents of PMMA.

The crystalline structure of PVT/PMMA composite films was characterized with X-ray diffraction (XRD). As shown in Figure 4, the peak at 19.8 belongs to (110, 200) of the β-phase [35]. With the increasing content of PMMA, the characteristic peak of the β-phase at 19.8 gradually disappeared. On the other hand, the unclear peaks at around 15° associated with the amorphous phase of PMMA widened, indicating that the crystallization of PVT was suppressed by the introduction of PMMA.

**Figure 5:**
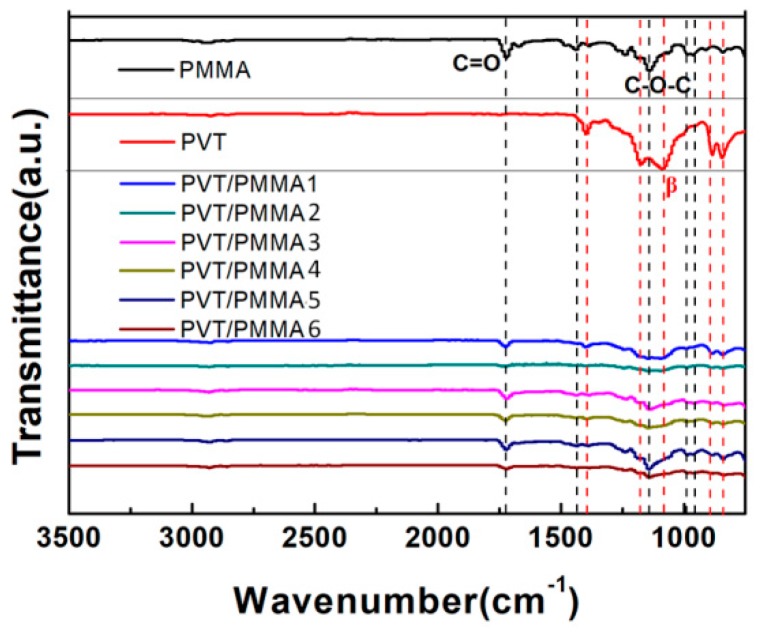
FTIR transmission spectra of pure P(VDF-TrFE), PMMA films, and PVT/PMMA composite films.

In order to further investigate the influence of PMMA on the conformation and crystallinity of P(VDF-TrFE), FTIR spectra were recorded between 400 and 4000 cm^−1^ for all samples, as shown in Figure 5. The absorption peaks at around 1150 cm^−1^ and 1740 cm^−1^ are attributed to the C-O-C and C=O stretching belonging to PMMA, respectively. These peaks appear in all the composite films except for pure P(VDF-TrFE) films, indicating the strong interactions between P(VDF-TrFE) and PMMA chains within the blends. The bands at 794, 880, 1175, 1210, and 1383 cm^−1^ correspond to the β-phase of P(VDF-TrFE), which represent the most characteristic bands, indicating that pristine P(VDF-TrFE) exists mainly in the β-phase. All characteristic peaks representing the β-phase of P(VDF-TrFE) gradually decreased with the addition of the PMMA component into the blended films, showing the decreasing crystallinity of the samples, which is consistent with the result of XRD.

### 3.2. Dielectric Properties

**Figure 6:**
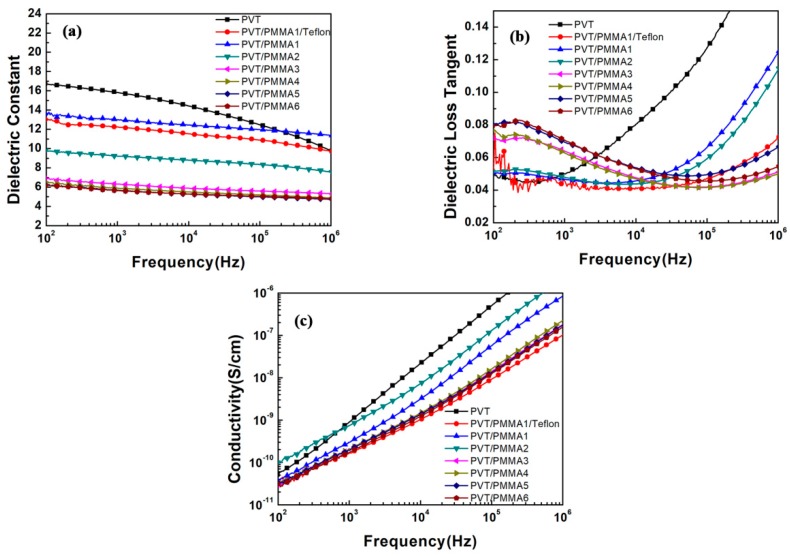
Frequency dependence of the (**a**) dielectric constant, (**b**) dielectric loss, and (**c**) electric conductivity of pristine P(VDF-TrFE) and PVT/PMMA blends at room temperature.

Figure 6a,b shows the dielectric properties of PVT/PMMA blended films over broad frequency ranges from 10^2^ Hz–10^6^ Hz. As the content of PMMA increases, the dielectric constant continuously decreases as a result of the dilution effect introduced by PMMA, which has a lower dielectric constant than pristine P(VDF-TrFE). The rigid PMMA segments also impede the shift of dipole groups in P(VDF-TrFE), restricting the dielectric response. In addition, the dielectric constant declines with increasing frequency (from 100 Hz–1 MHz). It can be explained that dipoles of the blends have enough time to reorient at lower frequencies, thereby presenting maximum dielectric values. As the frequency increases, the swing of dipoles does not have sufficient time to follow up with the external electric field, thus leading to a lower dielectric constant. Typically, Teflon-covered blended film with 20 wt % PMMA (PVT/PMMA1/Teflon) showed a dielectric constant of 13 at 100 Hz, which is a relatively high value in electrowetting application. From Figure 6b, it can be found that the dielectric loss of blended films increased with the increase of amorphous PMMA content. This phenomenon originates from the delayed reorientation of polymer chains, leading to higher heat dissipation. It is known that most glass-forming polymers have a loss peak at a certain range of frequencies, which is located at around 20–100 Hz for PMMA [36]. Therefore, at a frequency of around 100 Hz, blended films present increasing dielectric loss with the increase of amorphous PMMA content. However, at higher frequencies, dielectric loss significantly reduces with the addition of PMMA, because PMMA segments retard the relaxation of P(VDF-TrFE) in the blends. Figure 6c shows the electric conductivity of blended films over the frequency from 10^2^–10^6^ Hz. The electric conductivity of PVT/PMMA blended films remained extremely low (<10^−10^ S/cm) at 100 Hz, indicating the blended films have excellent insulation performance. Moreover, the electric conductivity of blended films showed strong dependence on frequency.

**Figure 7:**
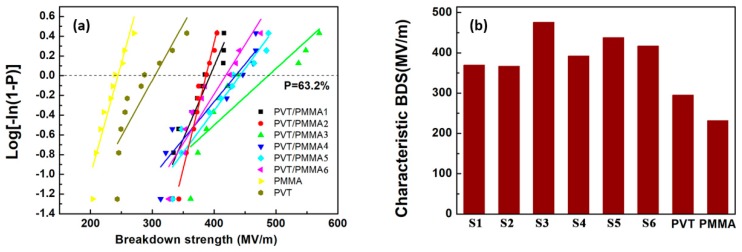
(**a**) Weibull plots of the breakdown strength for blended films and pristine films; (**b**) characteristic breakdown strength of blended films (S1: PVT/PMMA1, S2: PVT/PMMA2, S3: PVT/PMMA3, S4: PVT/PMMA4, S5: PVT/PMMA5, S6: PVT/PMMA6) and pristine films.

**Figure 8:**
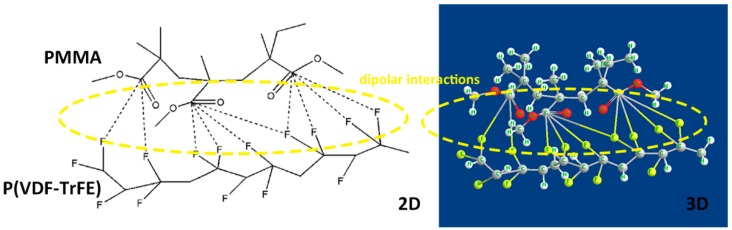
Schematic images of interactions among polar groups of PMMA and P(VDF-TrFE).

The dielectric breakdown strength (BDS) is also essential to dielectric films, determining the minimal thickness of insulator, maximum applied voltage and the number of voltage cycles in electrowetting application. Figure 7a,b displays the BDS Weibull distribution and characteristic breakdown strength of blended and pristine films at room temperature. It is worth noting that the breakdown strength of all blends is significantly higher than that of pure P(VDF-TrFE) and PMMA. It can be concluded that PMMA has strong interfacial interactions with P(VDF-TrFE), reducing the defect density in the film. The polar C-O-C and C=O groups belonging to PMMA can interact with strong polar fluoride groups of P(VDF-TrFE) (Figure 8). Besides, van der Waals forces and dipolar interactions in neighboring P(VDF-TrFE) and PMMA chains are introduced by the addition of PMMA [37]. Furthermore, the decreased crystal size and crystallinity [38] introduced by the incorporated PMMA segments could greatly suppress the possible free volume (or defect) existing in the normal P(VDF-TrFE) copolymers, thus improving the saturation electric field [39].

### 3.3. Hydrophobicity and Electrowetting Properties

**Figure 9:**
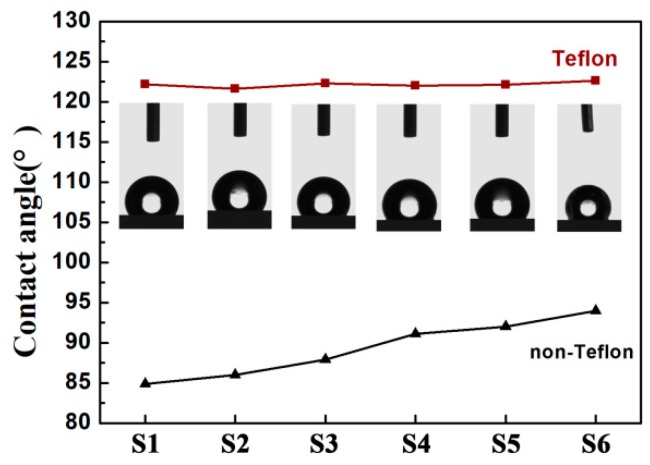
Static contact angle of bilayer dielectric (blended films covered with Teflon, PVT/PMMA/Teflon) and single-layer films (no Teflon covered, PVT/PMMA) in open air.

Generally, a large initial contact angle of the film is a crucial property for obtaining substantial electrowetting response under the electric field according to the Young–Lippmann equation. Figure 9 compares the initial static contact angles between bilayer dielectric (blended films covered with Teflon, PVT/PMMA/Teflon) and single-layer films (no Teflon covered, PVT/PMMA) in air. It can be obviously observed that the contact angle of films with no Teflon gradually increased as the concentration of PMMA increased. Such a phenomenon can be explained by the induced shift of the F atoms of P(VDF-TrFE) molecule chains, leading to the fluorine enrichment, as well as the hierarchical arrays at the interface. It is worth mentioning that all films covered with a layer of Teflon provided a significantly higher contact angle of over 120°, and there was no clear difference between different samples. The high initial contact angle is mainly attributed to the fluorine-containing (-CF_2_-) groups in the Teflon structure. Moreover, its special morphology, with polymer fibers randomly covering the surface, might also help to trap air and thus increase the contact angle [40].

**Figure 10:**
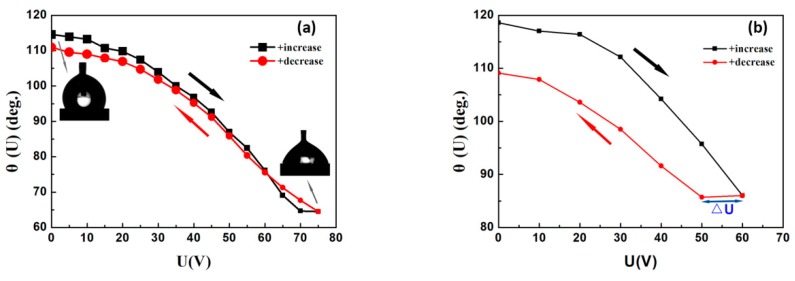
(**a**) Contact angle modulation for water in air on PVT/PMMA1/Teflon at AC voltages from 0–75 V; (**b**) contact angle modulation for water in air on PVT/PMMA1/Teflon at DC voltages from 0–60 V.

Both AC and DC voltages were applied to the PVT/PMMA1/Teflon bilayer to observe the contact angle modulation and hysteresis of a water droplet in air. As shown in Figure 10a, the contact angle changed from 115°–65° (Δ*θ* = 50°) with an external AC voltage change from 0–75 V. It was also reported that the initial contact angle could reach 165° in a silicone oil ambient condition, because of the lower surface tension ($\gamma_{water-oil}$: 0.038 N/m), approximately half of that by air ($\gamma_{water-air}:$ 0.0728 N/m) [30]. Therefore, a contact angle modulation of 50° represents a high tuning range for EWOD applications in open air. Reversibility is another important property for a suitable dielectric for practical EWOD-based devices. For many ferroelectric polymers, large CAH is the main reason that restricts their applications. Here, P(VDF-TrFE) modified with 20 wt % PMMA and coated with a thin layer of Teflon with low roughness shows excellent reversibility at AC voltage.

Positive DC voltage was also applied to the bilayer dielectric. Figure 10b shows that the contact angle modulation decreased with the increase of DC voltage at the rate (dU/dt) of 10 V/s. Meanwhile, there was a hysteresis and corresponding offset voltage Δ*U* ≈ 10 V, which can be attributed mainly to the induced remnant polarization of ferroelectric P(VDF-TrFE). It is well-known that the nature of ferroelectric polymers induces irreversible polarization. As an external electric field is applied, the dipoles align along the electric field direction. However, only a small portion of the dipoles can reverse to the original position once the electric field is removed, leading to remnant polarization. The addition of PMMA can reduce the ferroelectric property of P(VDF-TrFE), and thus weaken, but not eliminate, its influence on DC electrowetting. Moreover, it has been demonstrated that the hysteresis could be further minimized when the medium of air is replaced by oil, due to the smoother motion of droplets in oil and lower interfacial tension [20,41].

As mentioned above, the dielectric constant (*ε*), initial contact angle (*θ*_0_), and the breakdown field value (E_BD_) are all critical material parameters for low-voltage EWOD applications. The voltage required to achieve desirable contact angle modulation (Δ*θ*) of a water droplet ($\gamma_{LV}$, *L*-water; *V*-air) on dielectric film ($\varepsilon_{0}\varepsilon$) is given by:

(2) $$U^{2}=\frac{2\gamma_{LV}\left[\cos\theta \left(U\right)-cos\theta_{0}\right]}{\varepsilon_{0}\varepsilon }d$$

**Figure 11:**
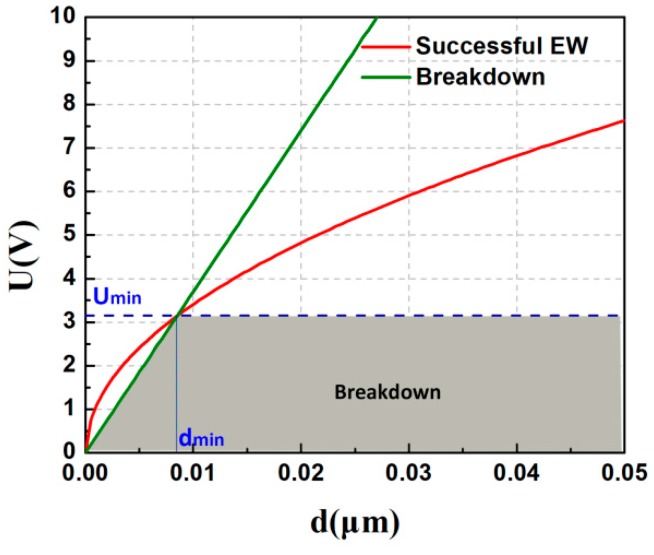
Effective electrowetting and dielectric breakdown voltage versus insulator thickness. Green solid line: critical voltage for dielectric breakdown (for EBD = 370 V/μm). Red solid line: voltage required for a contact angle decrease from 120°–60° (for $\gamma_{\mathrm{LV}}$ = 0.0728 N/m between water and air; ε = 13). The dashed line indicates the minimum applied voltage and insulator thickness.

Dielectric breakdown occurs at *U*_BD_ = *E*_BD_*d*. The competition between these two effects (effective electrowetting and breakdown) is illustrated in Figure 11. The intersection between the straight line (green) and the square root function (red) determines the minimum thickness at certain Δ*θ* for a given permittivity, also implying the corresponding lowest voltage [1]. As for PVT/PMMA1/Teflon, the dielectric constant (*ε*) is 13 at 100 Hz, and the breakdown strength (*E*_BD_) is around 370 V/μm. Thus, as is shown in Figure 11, the theoretical minimum voltage for effective electrowetting on PVT/PMMA1/Teflon film can be as low as 3.1 V in air. The limitation of this procedure is that, because the real dielectric strength of thin layers may differ from the calculated value, there may be a bias between theoretical and experimental results. However, this conclusion still indicates that such a bilayer composed of PVT/PMMA1 and Teflon is a promising dielectric for low-voltage EWOD applications.

### 3.4. Energy Storage

**Figure 12:**
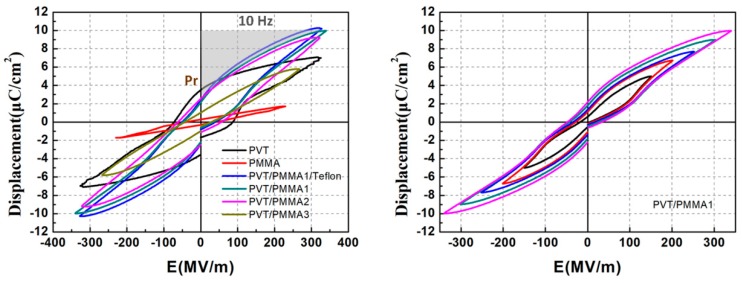
(**a**) D-E hysteresis loops of blended films, pristine P(VDF-TrFE), and PMMA film; (**b**) D-E hysteresis loops of PVT/PMMA1 film at various electric fields.

Finally, D-E hysteresis loops’ measurements were performed to investigate the effect of PMMA on the ferroelectric properties and energy density of P(VDF-TrFE). Figure 12a compares the bipolar D–E loops of the blended films with varied PMMA content under AC electric field at 10 Hz. The D–E loops turn from a rectangular shape into a linear shape gradually with the increasing content of PMMA. The rectangular shape of D-E loops is attributed to the remnant polarization (*P*_r_) as explained above. The reduction of remnant polarization confirms that PMMA restricts the ferroelectric phase of P(VDF-TrFE). Interestingly, the maximum displacement of PVT/PMMA1 is higher than pristine P(VDF-TrFE), reaching 10.1 μC/cm^2^, which is significantly higher than that of Ref. [36] (4.7 μC/cm^2^). That may be attributed to the decreased free volume compared to normal P(VDF-TrFE), since the polarization of air voids is utterly negligible compared to that of polymers. The enhanced interfacial interaction among polar groups and neighboring chains of blend components may also contribute to the improved polarization. It is understandable that the D–E loop of PVT/PMMA1/Teflon is almost the same as PVT/PMMA1, because the thickness of the Teflon layer is negligible compared to that of the PVT/PMMA blend layer. The D-E loops of PVT/PMMA1 in Figure 12b growing parallel with the electric field without shrinking further as a function of E demonstrate that the content of PVT/PMMA1 is stable. Besides, the energy storage density of PVT/PMMA1 is around 11.8 J/cm^3^, which can be calculated by integrating the absolute area of P-E loops as seen in Figure 12a, that is U=∫EdD, where U is the energy density, *E* is the electric field, and *D* is the displacement, while the energy density of pristine P(VDF-TrFE) is only 6.1 J/cm^3^. Significant enhancement of energy density is achieved in blended films compared to the pristine P(VDF-TrFE) film. Therefore, PVT/PMMA blended film containing 20 wt % PMMA also shows great potential for industrial application for polymeric energy storage capacitors.

## 4. Conclusions

In summary, a significantly promising blended film synthesized in this paper showed outstanding performances for practical electrowetting and high energy-density applications. The addition of PMMA affected the crystallinity of P(VDF-TrFE), including the dilution and impeding the influence on the overall crystallization of ferroelectric phase. The van der Waals forces and dipolar interactions strengthened the interfacial interactions, while suppression of free volume (or defect) was essential to the significant enhancement of breakdown strength and energy storage. The typical, Teflon-covered PVT/PMMA blend containing 20 wt % PMMA exhibited a high dielectric constant of 13 and an initial contact angle of 122°. Further electrowetting responses with a contact angle modulation of 50° in air and low CAH demonstrated that this material is promising for low-voltage electrowetting applications. Furthermore, the PVT/PMMA blend containing 20 wt % PMMA with significantly-enhanced energy storage capability even turned out to be a superior material for capacitor applications.
